# Hyperoside inhibits the effects induced by oxidized low-density lipoprotein in vascular smooth muscle cells via oxLDL-LOX-1-ERK pathway

**DOI:** 10.1007/s11010-017-3025-x

**Published:** 2017-04-22

**Authors:** Zhengyu Zhang, Dongdong Zhang, Baoling Du, Zhiqiang Chen

**Affiliations:** 1 Guangzhou Institute of Cardiovascular Disease, The Second Affiliated Hospital of Guangzhou Medical University, Guangzhou, 510260 China; 2 Department of Cardiology, Guangzhou Medical University, Guangzhou, 511436 China; 3 Department of Ultrasonic, Liaocheng People’s Hospital, Liaocheng, 252000 China; 4 Department of Orthopedics, Liaocheng People’s Hospital, Liaocheng, 252000 China

**Keywords:** Atherosclerosis, Hyperoside, oxLDL, LOX-1, Receptor, Cell signaling

## Abstract

Hyperoside is a major active constituent in many medicinal plants traditionally used in Chinese medicines for their anti-inflammatory, antioxidative, and vascular protective effects. Recent studies have focused on the protective effects of hyperoside on hyperlipidemia. However, the molecular mechanisms underlying these effects are unknown. In this study, vascular smooth muscle cells (VSMCs) were treated in vitro with oxidized low-density lipoprotein (oxLDL) in the presence or absence of hyperoside. Western blotting, quantitative PCR, and tetrazolium assay were used to detect lectin-like oxLDL receptor-1 (LOX-1) expression and extracellular signal-regulated kinases (ERK) activation, and to determine VSMCs viability. The results demonstrated that oxLDL promoted LOX-1 expression, ERK activation, and proliferation in VSMCs. Hyperoside significantly inhibited the oxLDL-stimulated effects after long time exposure. However, it promoted ERK activation directly following a short incubation duration (25 min). In conclusion, hyperoside inhibits oxLDL-induced LOX-1 expression, ERK activation, and cell proliferation through the oxLDL-LOX-1-ERK pathway in VSMCs. Our findings suggest a novel role of hyperoside in treating and preventing atherosclerosis.

## Introduction

Hyperoside is a flavonoid compound mainly found in herbal plants traditionally used in Chinese medicines and has many biological effects. Accumulating evidence suggests that hyperoside has neuroprotective, anti-cancer, anti-inflammatory, antioxidative, and vascular protective effects [1–6]. However, the molecular mechanism underlying these effects is unknown.

Oxidation and inflammation both play important roles in the development of atherosclerosis and vascular remodeling. These effects are all involved in oxidized low-density lipoprotein (oxLDL)-induced vascular damage. In patients with hyperlipidemia, oxLDL can combine with scavenger receptors on the surface of vascular smooth muscle cells (VSMCs); lectin-like oxLDL receptor-1 (LOX-1) is one of the most important scavenger receptors on VSMCs. It is also well known that oxLDL promotes VSMC proliferation by activating extracellular signal-related kinases (ERK) [7–9]. Therefore, oxLDL-LOX-1-ERK pathway inhibition is important in the development of atherosclerosis and cardiovascular remodeling.

Several studies have reported the effect of hyperoside on LDL. Extracts from the leaves and flowers of *Crataegus aronia* that contain hyperoside decreased the serum total cholesterol and LDL of hypercholesterolemic rats [10]. In addition, hyperoside prolonged the lag time in conjugated diene formation in Cu^2+^-induced LDL in vitro [11]. These results indicate that flavonoids, including hyperoside, can not only decrease LDL levels but also inhibit LDL oxidation [10–14].

In the study of cellular signaling, we previously found that hyperoside attenuated ECV304 cell damage induced by advanced glycation end products [15]. Similarly, other studies reported that hyperoside protected cells by inhibiting oxidative stress, stimulating or inhibiting mitogen-activated protein kinase (MAPK) activation; these effects were direct or indirect [1–3, 6, 15–17]. For most of the indirect effects, modifying receptor expression is an important pathway. For example, hyperoside inhibited both VSMC proliferation in vitro and carotid artery ligation-induced neointimal formation in vivo by inducing Nur77 expression and activation [1]. Chu et al. reported that extract of *Zanthoxylum ailanthoides* that included rutin and hyperoside decreased the expression of scavenger receptor class AI (SR-AI) and CD36 [12]. However, there has been no report of the effect of hyperoside on the expression of LOX-1, an important receptor in oxLDL-induced atherosclerosis.

We believe that hyperoside has a protective effect against hyperlipidemia, but its molecular mechanism is largely unknown. Therefore, we carried out this study to elucidate whether hyperoside inhibits ERK activation and VSMC proliferation by downregulating LOX-1.

## Materials and methods

### VSMC culture

VSMCs were isolated by enzymatic digestion of the aortas of C57BL/6J mice using a modified version of a procedure described previously [18]. The isolated cells were cultured in Dulbecco’s modified Eagle’s medium (DMEM; Life Technologies, Carlsbad, CA, USA) supplemented with 10% fetal calf serum, penicillin, and streptomycin at 37 °C in a humidified atmosphere of 5% CO_2_. The medium was changed every 2 days, and cells were passaged by treatment with 0.05% trypsin–0.02% EDTA solution. Experiments were conducted on VSMCs that had just achieved confluence. Cells between passages 5 and 15 were used; immunofluorescence staining and western blotting determined that the cells expressed smooth muscle α-actin.

All animal work has been conducted according to relevant national and international guidelines. Institutional Animal Care and Use Committee of Guangzhou Medical University has approved this research.

### Western blotting

The western blotting procedures used were similar to that described previously with slight modifications [7]. Treated VSMCs were harvested in lysis buffer using protease inhibitors. The lysate suspension was centrifuged and protein concentration was assessed using a Bio-Rad protein assay. Heat-denatured proteins were resolved by sodium dodecyl sulfate-polyacrylamide gel electrophoresis (SDS-PAGE) and electrophoretically transferred onto nitrocellulose membranes. These were probed with antibodies against phosphorylated ERK (P-ERK; Cell Signaling Technology, Danvers, MA) and LOX-1 (Santa Cruz Biotechnology, Santa Cruz, CA) and re-probed with β-actin or pan-ERK antibody (Cell Signaling Technology, Danvers, MA). The bands were visualized using an enhanced chemiluminescence (ECL) detection system (GE Healthcare, Piscataway, NJ); intensity was quantitated using densitometry.

### Real-time quantitative PCR (qPCR)

The qPCR was performed similarly to procedures described previously [15]. In brief, it was performed with Power SYBR Green PCR Master Mix (Takara Biotechnology, Dalian, China) on an ABI 7900 Real-Time PCR instrument according to the manufacturer’s instructions (Applied Biosystems, Foster City, CA). The primer sequences used were designed using Primer 3 (Whitehead Institute/MIT, Cambridge, MA) and confirmed by BLAST in GenBank. The forward (F) and reverse (R) qPCR primers for amplifying the LOX-1 [NM_001301096] and glyceraldehyde-3-phosphate dehydrogenase (GAPDH) complementary DNA (cDNA) were synthesized by Takara Biotechnology as follows: GAPDH: F-5′-TGTGTCCGTCGTGGATCTGA-3′ and R-5′-TTGCTGTTGAAGTCGCAGGAG-3′; LOX1: F-5′-TGAAGCCTGCGAATGACGAG-3′ and R-5′-GTCACTGACAACACCAGGCAGAG-3′.

### RNA interference

The procedures used for this experiment were similar to that described in our previous report [7]. The LOX-1 small interfering RNA (LOX-1-siRNA) target duplex sequences were synthesized by Invitrogen (Carlsbad, CA). A non-targeting siRNA duplex sequence (Stealth™ RNAi, Invitrogen, Carlsbad, CA) was used as the negative control (NC). VSMCs were transfected using 100 pmol of each siRNA duplex per well using Lipofectamine 2000 (Invitrogen, Carlsbad, CA) and Opti-MEM (Invitrogen, Carlsbad, CA) according to the manufacturer’s recommendations. After 24-h transfection, the cells were serum-starved for an additional 24 h and then harvested to detect ERK phosphorylation by western blotting.

### Tetrazolium (MTT) cell viability assay

Cell viability was determined using a CellTiter 96 Non-Radioactive Cell Proliferation Assay kit (Promega, Madison, WI) according to the manufacturer’s instructions. Briefly, VSMCs were seeded in 96-well culture plates at 3000 cells/well. After incubation at 37 °C for 24 h, the medium was replaced with fresh medium supplemented with or without hyperoside (50 μg/ml) (Sigma Chemical Co., St. Louis, MO). After 10-min incubation, oxLDL (25 μg/ml) was added to the cells. Dye solution was added to each well 48 h after treatment and the cells were incubated at 37 °C for 2 h. Solubilization/stop solution was added to each well and the absorbance was measured at 570 nm using an enzyme-linked immunosorbent assay reader. Relative cell numbers were calculated after normalizing the absorbance to untreated cells. Cell viability was calculated relative to untreated cells.

### Statistical analysis

Data from three independent experiments are expressed as the mean ± SD. Statistical analyses were performed with one-way analysis of variance (ANOVA). *P*-values below 0.05 were considered significant.

## Results

### Hyperoside downregulates oxLDL-induced LOX-1 expression in VSMCs

**Fig. 1:**
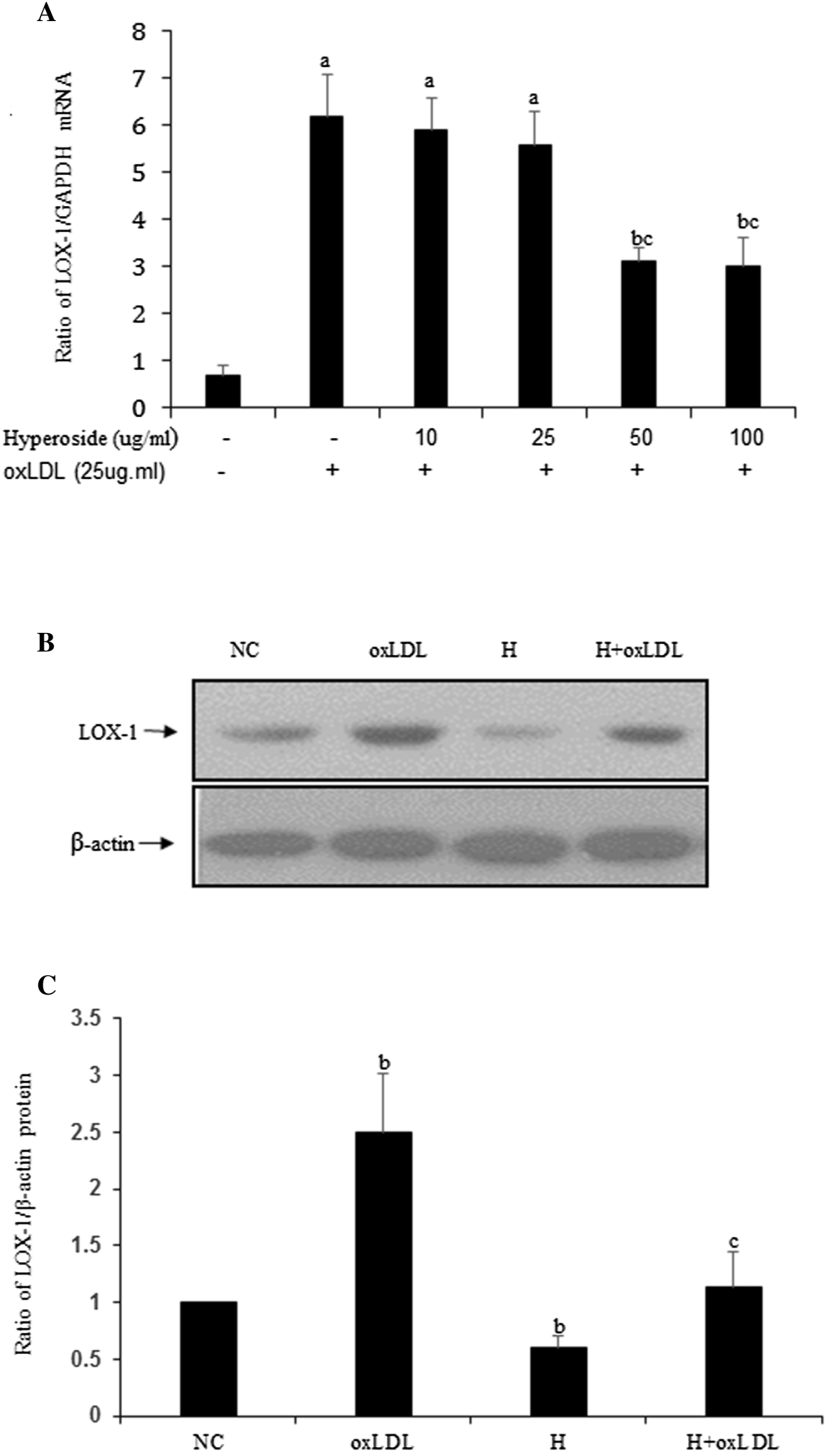
Hyperoside downregulates oxLDL-induced LOX-1 expression in VSMCs. **a** qPCR shows *LOX*-*1* expression levels in VSMCs treated with oxLDL in different concentrations of hyperoside for 12 h. **b** Western blot shows LOX-1 expression levels in VSMCs treated with oxLDL in the absence or presence of hyperoside for 24 h. **c** The statistical results of (**b**) are (*a*) versus NC (*P* < 0.01), (*b*) versus NC (*P* < 0.05), and (*c*) versus oxLDL (*P* < 0.05); results are from three independent experiments. *NC* negative control, *H* hyperoside

To determine whether hyperoside could affect LOX-1 expression induced by oxLDL, quiescent VSMCs were treated with oxLDL with or without hyperoside. Real-time qPCR (Fig. 1a) showed that with the concentration of 50 and 100 μg/ml, hyperoside significantly inhibited oxLDL-induced *LOX*-*1* upregulation. Then hyperoside (50 μg/ml) was used in western blotting (Fig. 1b, c). The results showed that in the absence of hyperoside, oxLDL significantly increased LOX-1 expression relative to the NC (*P* < 0.05). In contrast, hyperoside significantly inhibited this effect (*P* < 0.05). These results suggest that oxLDL may induce LOX-1 upregulation and that hyperoside inhibits this effect.

### OxLDL promotes cell viability and hyperoside inhibits these effects

**Fig. 2:**
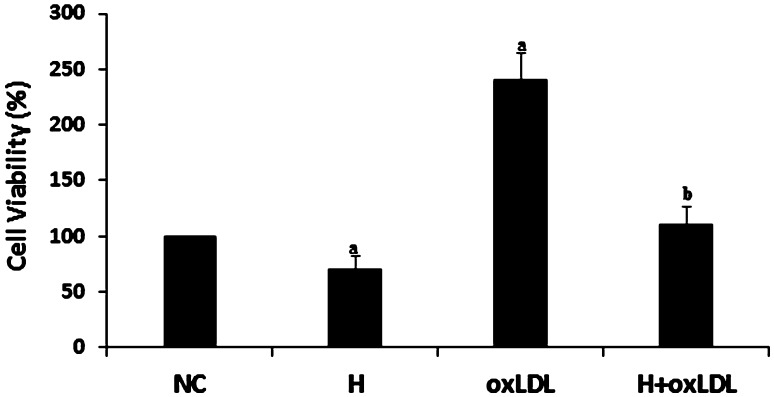
Hyperoside inhibits cell viability and attenuates the effects of oxLDL. Cells were pretreated with or without hyperoside (50 μg/ml). After 10-min incubation, oxLDL (25 μg/ml) was added to the cells. Dye solution was added to each well 48 h after treatment and the cells were incubated at 37 °C for 2 h. Results shown are a percentage of the NC, (*a*) versus NC (*P* < 0.05) and (*b*) versus oxLDL (*P* < 0.05). *NC* negative control, *H* hyperoside

OxLDL promotes VSMC proliferation [19, 20]. In this study, we detected the effect of hyperoside on oxLDL-stimulated cells by MTT assay. The cell viability between the oxLDL-treated and NC groups was significantly different (*P* < 0.05), indicating that oxLDL promoted cell viability in VSMCs. The viability of cells treated with oxLDL and hyperoside was significantly lower relative to cells treated with oxLDL alone (*P* < 0.05; Fig. 2), indicating that hyperoside inhibits oxLDL-induced cell viability. We also found that hyperoside alone inhibited cell viability compared to the NC.

### Hyperoside cannot directly inhibit oxLDL-induced ERK activation

**Fig. 3:**
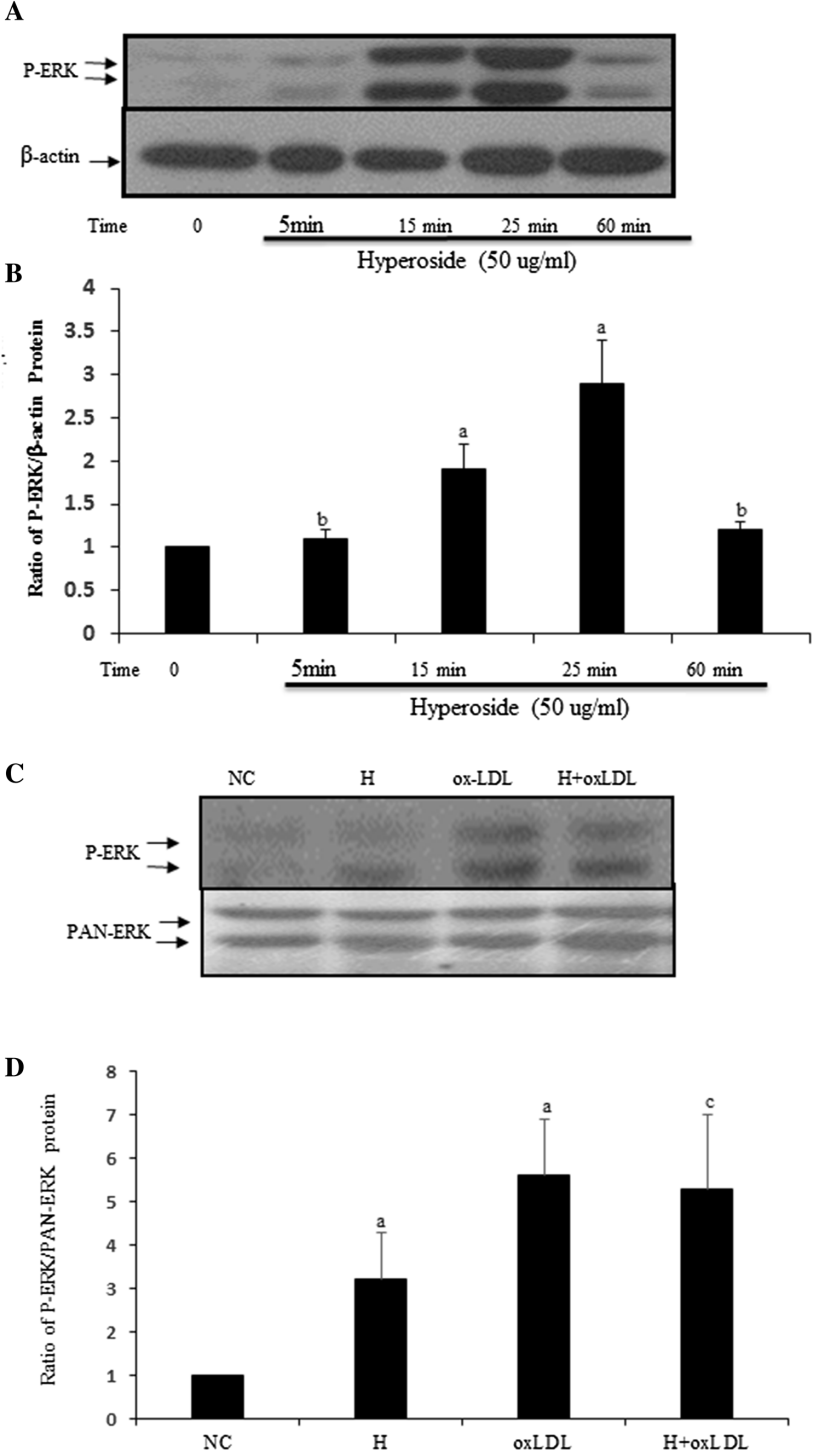
Hyperoside does not inhibit oxLDL-induced ERK activation in VSMCs directly. **a** Cells were treated with hyperoside from 5 min to 1 h; **b** cells were pretreated with hyperoside and then treated with oxLDL. Western blot shows P-ERK activation. **b**, **d** the statistical results of (**a**, **c**) are (*a*) versus NC (*P* < 0.05), (*b*) versus NC (*P* > 0.05) and (*c*) versus oxLDL (*P* > 0.05). Results are from three independent experiments. *NC* negative control, *H* hyperoside

To date, no data are available on the effects of hyperoside in oxLDL-induced ERK activation in VSMCs. P-ERK is activated ERK; more P-ERK can be detected when certain stimuli activate ERK. Western blotting (Fig. 3) showed that oxLDL induced ERK activation. No significant alteration was detected when VSMCs were incubated with oxLDL and hyperoside (*P* > 0.05). Compared to the NC, hyperoside significantly promoted ERK activation and reached peak after 25-min incubation. These results suggest that hyperoside may not have a direct inhibitory effect on oxLDL-induced ERK activation.

### Hyperoside inhibits oxLDL-induced ERK activation by downregulating VSMC LOX-1 expression

**Fig. 4:**
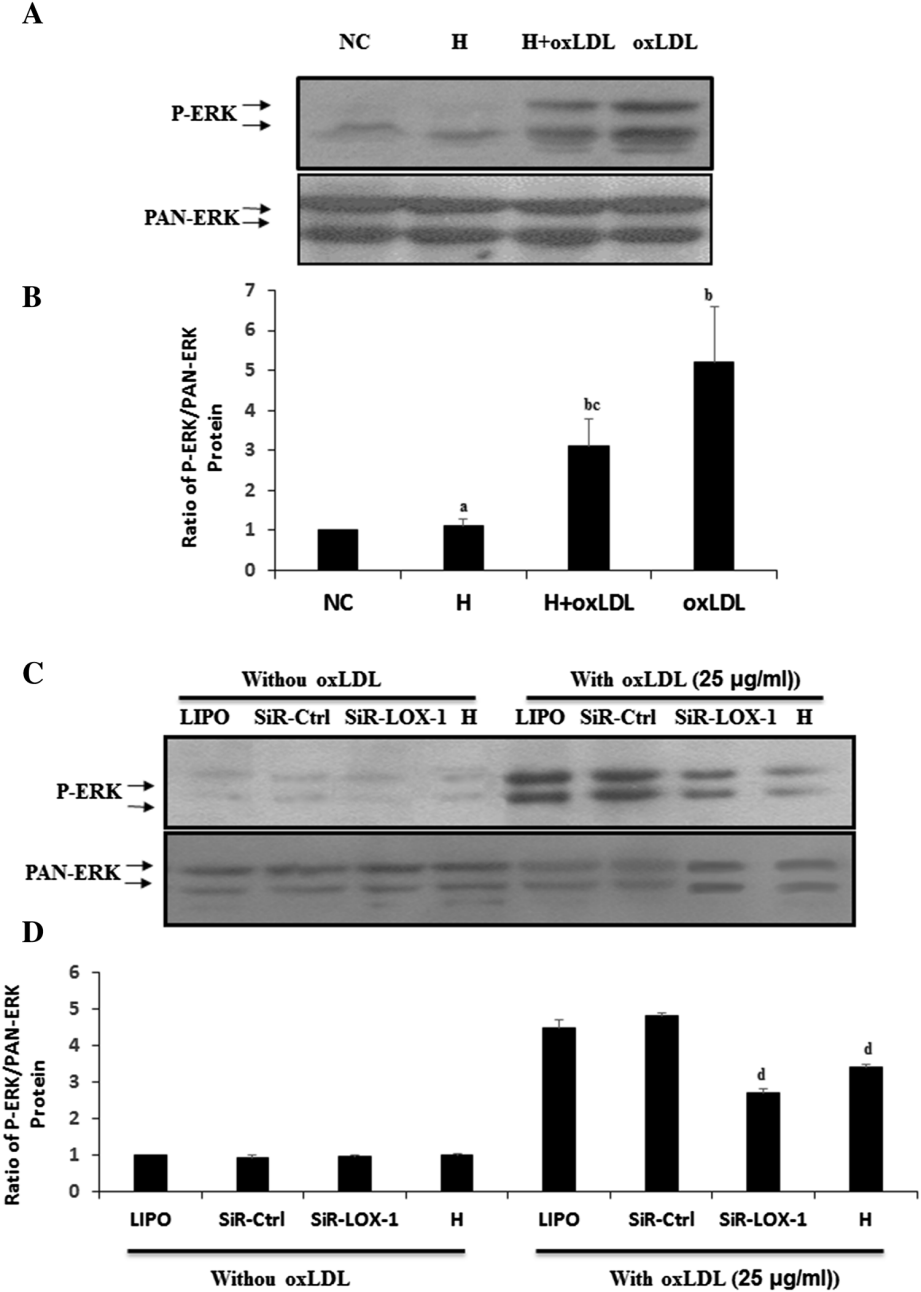
Hyperoside inhibits oxLDL-induced ERK activation by downregulating LOX-1 expression in VSMCs. **a** VSMCs were pretreated with or without hyperoside and then treated with oxLDL. Western blot shows the ERK activation. **c** Cells were pretreated with LOX-1-siRNA or hyperoside, serum-starved, and then treated with oxLDL. Western blotting detected ERK activation. **b**, **d** The statistical results of (**a**, **c**) are (*a*) versus NC (*P* < 0.05) and (*b*) versus oxLDL (*P* < 0.05). Results are from three independent experiments; *LIPO* Lipofectamine 2000, *H* hyperoside

If hyperoside could not inhibit ERK activation, how could it inhibit oxLDL-induced cell viability? To elucidate the effect of hyperoside on the oxLDL-LOX-1-ERK signaling pathway, cells were treated with or without hyperoside for 24 h, and then treated with oxLDL for 15 min before harvesting for western blotting to detect ERK activation. Western blotting (Fig. 4a, b) showed significantly decreased ERK activation in VSMCs pretreated with hyperoside compared with oxLDL group (*P* < 0.05). Compared with NC group, no significant alteration was detected when VSMCs were incubated with hyperoside for 24 h. These results suggested that hyperoside may inhibit oxLDL-induced ERK activation by altering the expression of oxLDL receptors. To confirm this hypothesis, cells were pretreated with LOX-1-siRNA or hyperoside for 48 h, and then treated with oxLDL for 15 min before harvesting. Western blotting (Fig. 4c, d) showed that ERK activation in cells pretreated with LOX-1-siRNA and hyperoside was inhibited. No differences were detected in the groups not treated with oxLDL. These results indicate that hyperoside has the similar function as LOX-1-siRNA in inhibiting the expression of LOX-1.

## Discussion

This study demonstrated that hyperoside has protective effects against oxLDL-induced LOX-1 expression, ERK activation, and proliferation in VSMCs. First, hyperoside suppresses LOX-1 expression at gene and protein level. Second, it inhibits VSMC viability. Third, it directly promotes ERK activation following short-duration incubation (25 min), but suppresses oxLDL-induced ERK activation by downregulating LOX-1 expression, and eventually suppresses cell proliferation.

Hyperoside has anti-inflammatory, antioxidative, and vascular protective effects [1, 21–23]. Accordingly, we detected its effect on oxLDL-induced LOX-1 expression. As expected, hyperoside suppressed LOX-1 expression at gene and protein level (Fig. 1). Next, we detected cell viability by incubating VSMCs with hyperoside in the absence or presence of oxLDL, and found that hyperoside inhibits VSMC viability (Fig. 2). This result is similar to that of another report [1]. However, the signaling pathway of these effects is unknown.

Recent findings have demonstrated that hyperoside promotes ERK activation in a time- and dose-dependent manner. ERK is the downstream signaling molecule of oxLDL-LOX-1 [7, 24]. In this study, 25-min treatment with hyperoside promoted ERK activation. However, there were no significant differences in ERK activation in cells treated with hyperoside and oxLDL together as compared to oxLDL-treated cells. The ERK activation of the two groups was significantly higher than that of the NC (Fig. 3). These results indicate that hyperoside promotes ERK activation alone and cannot inhibit oxLDL-induced ERK activation. We also did not find a synergistic effect between hyperoside- and oxLDL-induced ERK activation (Fig. 3). However, this was a short-duration study. When cells were pretreated with hyperoside for 24 h and then incubated with oxLDL for 15 min, we obtained wholly different results in that hyperoside pre-treatment inhibited oxLDL-induced ERK activation (Fig. 4a, b).

The short-duration promoting effect of hyperoside on ERK activation should promote cell proliferation or viability, while our study indicated that hyperoside suppresses oxLDL-induced promotion of cell viability (Fig. 2). There are two possible reasons for this: one is that hyperoside triggered other signaling pathways that induce apoptosis. Some have reported the effect of hyperoside on P38 [16, 25]. The other possible reason is that hyperoside alters the expression of some receptors on the oxLDL-related signaling pathway. In our study, we found that hyperoside suppressed the expression of LOX-1, one of the most important receptors on the oxLDL signaling pathway. Therefore, lower LOX-1 expression decreased formation of the oxLDL–LOX-1 complex, attenuating the effects of oxLDL and suppressing ERK activation and cell viability (Figs. 2, 4a, b). Other studies have also published similar reports in that hyperoside inhibited the expression of Nur77, SR-AI, and CD36 [1, 12].

To further confirm our findings, cells were incubated with LOX-1-siRNA or hyperoside, and then treated with oxLDL. Previously, we reported that siRNA inhibits ERK activation [7]. In the present study, hyperoside inhibited ERK activation, a similar effect to that of siRNA (Fig. 4c, d). These results indicate that hyperoside at least partially inhibits the oxLDL-LOX-1-ERK signaling pathway by suppressing LOX-1 expression.

**Fig. 5:**
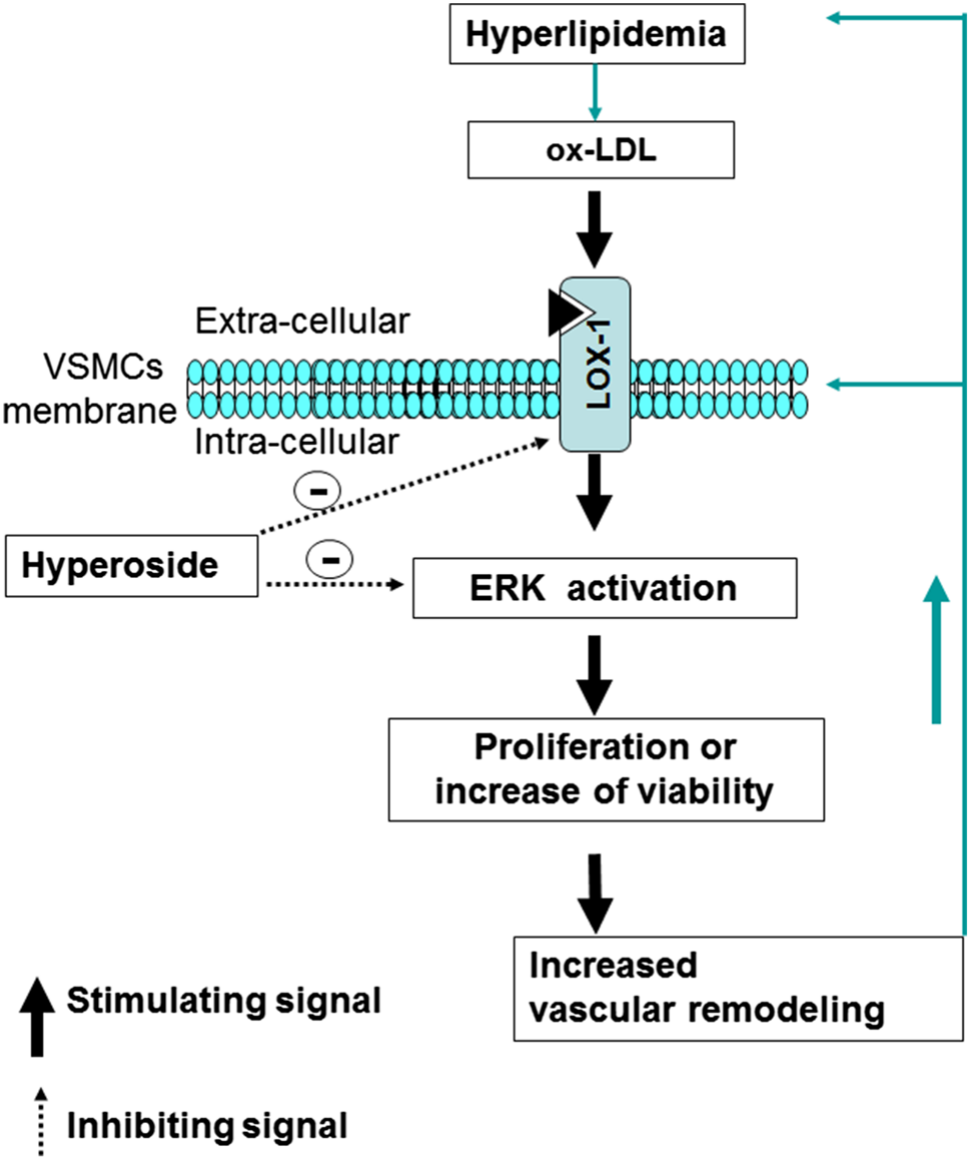
Diagram shows the effect of hyperoside on the oxLDL-LOX-1-ERK signaling pathway and VSMC proliferation. Increased blood LDL can trigger oxLDL increases in the walls of vein grafts and arteries. As a LOX-1 ligand, oxLDL can activate LOX-1 and its downstream signaling molecules, including ERK. Activated ERK leads to LOX-1 overexpression and promotes VSMCs viability, thereby altering vascular structure and function. Blocking LOX-1 and its downstream signaling molecules by hyperoside might inhibit vascular remodeling induced by oxLDL

In conclusion, we illustrate a novel role of hyperoside in inhibiting oxLDL-induced ERK activation and cell proliferation in VSMCs through the oxLDL-LOX-1-ERK pathway (Fig. 5). These findings might advance current understanding of the effects of hyperoside on vascular protection.
